# Urology boot camp for medical students: Using virtual technology to enhance undergraduate education

**DOI:** 10.1002/bco2.213

**Published:** 2023-02-09

**Authors:** Thomas Fonseka, Mei‐Ling Henry, Clare Ellerington, Arjun Gowda, Ricky Ellis

**Affiliations:** ^1^ Department of Urology Royal Derby Hospital, University Hospitals of Derby and Burton Derby UK; ^2^ Department of Medical Education, Royal Derby Hospital University Hospitals of Derby and Burton Derby UK

**Keywords:** online learning, simulation, surgical training, undergraduate medical education, urology boot camp, virtual technology

## Abstract

**Objectives:**

The study aims to describe the methodology of converting the urology boot camp for medical students into a virtual course with key take home points for a successful conversion and to present quantitative and qualitative data demonstrating the impact of the boot camp on improving delegates' knowledge and clinical acumen.

**Materials and methods:**

The face‐to‐face boot camp was converted to a virtual format employing a variety of techniques including; utilizing an online platform to deliver live screened lectures, using online polling software to foster an interactive learning environment and displaying pre‐recorded videos to teach practical skills. Validated Multiple Choice Questionnaires (MCQs) were used prior to and after the course. This enabled the assessment of delegates' knowledge of urology according to the national undergraduate curriculum, and paired *t* tests were used to quantify the level of improvement. Thematic analysis was carried out on post‐course delegate feedback to identify highlights of the course and ways of improving future iterations.

**Results:**

In total, 131 delegates took part in the pilot virtual course. Of these, 105 delegates completed the pre‐ and post‐course MCQs. There was a statistically significant improvement in the assessment following the course (*p* = <0.001) with mean score increasing from 47.5% pre‐course to 65.8% post‐course. All delegates who attended the most recent implementation of the virtual course (*n* = 31) felt it improved their knowledge and confidence in urology. Twenty delegates (64.5%) felt that it prepared them for both final year medical school examinations and working as a foundation year doctor. Positive themes in feedback were identified, which included the interactive nature of the course, the quality of teaching, the level and content of information provided and the high yield, concise organization of the teaching schedule.

**Conclusion:**

Using virtual technology and innovative educational frameworks, we have demonstrated the successful conversion of the urology boot camp for medical students to a virtual format. At a national level, with support from the British Association of Urological Surgeons, the face‐to‐face component of the course will continue to run in parallel with the virtual course with the aim of standardizing and improving UK undergraduate urological education. The virtual course has been implemented on an international scale, and this has already shown promising results.

## 1 INTRODUCTION

**FIGURE 1:**
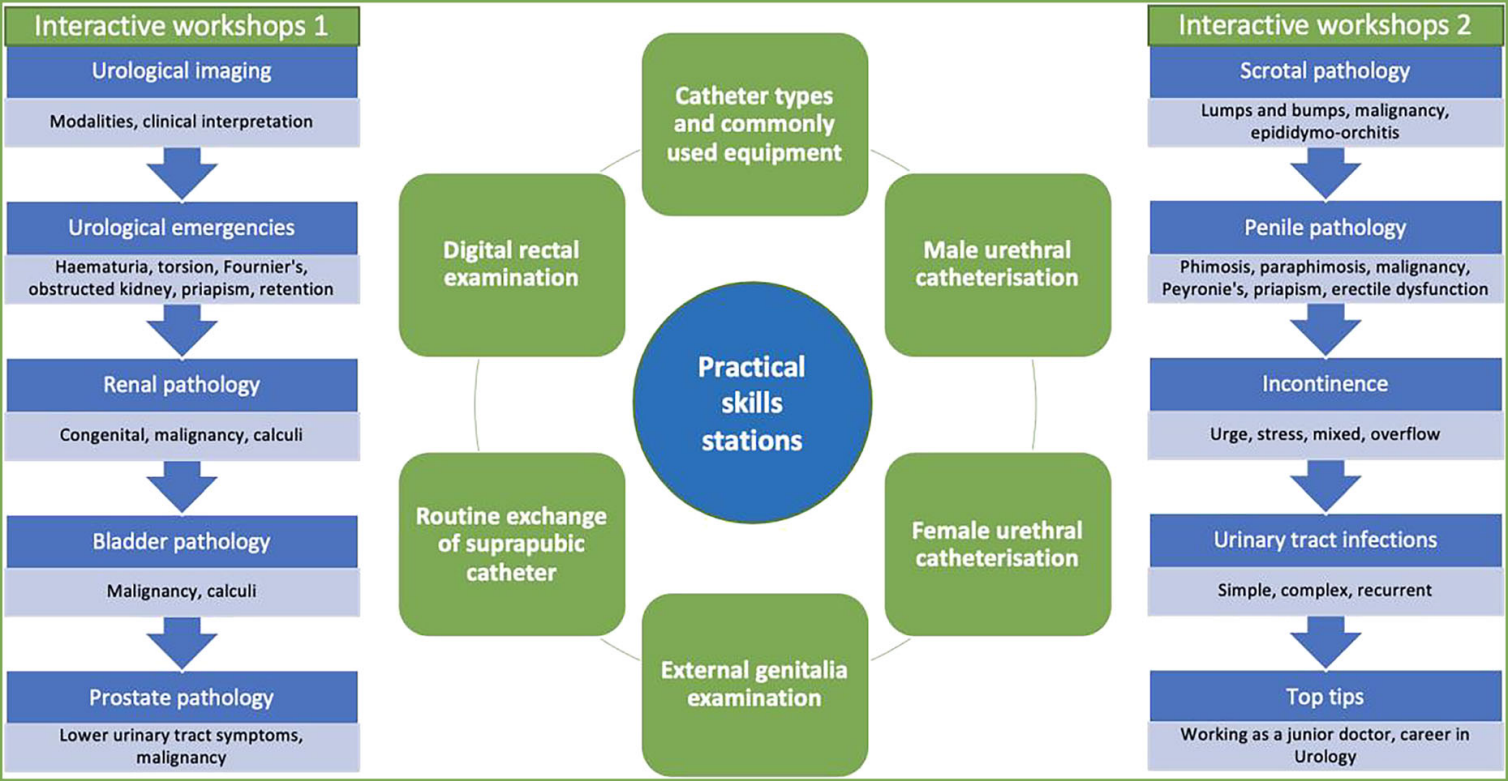
Schematic representation of the theory‐based and clinically oriented topics covered through both interactive workshops and practical skills stations. Practical skills stations incorporate simulation models and case‐based discussions.

The coverage of and exposure to urology in medical school curricula across the UK is often variable and sometimes lacking.^1^ This is despite urological conditions accounting for over 25% of acute surgical referrals and 10%–25% of all general practitioner appointments.^2^ The urology boot camp for medical students is a registrar‐led 1‐day course of compact, high‐intensity learning that covers the entire undergraduate urology curriculum.^2^, ^3^ The core components of the course are displayed in Figure 1. It combines theory‐based learning with practical skills with the aim of equipping delegates with the knowledge and clinical acumen to pass their medical school final examinations and be competent in managing urological conditions as foundation year doctors. The urology boot camp for medical students has been running for over 5 years now and has become an integral course for medical students at the University Hospitals of Derby and Burton, UK. Its effectiveness and high yield for educational value has been previously published, with excellent results in improving knowledge, skills and confidence.^3^ Part of what has made this course successful and popular amongst medical students has been its focus on interactive teaching, hands‐on practical skills and group learning.

Unfortunately, the COVID‐19 pandemic led to numerous teaching courses, like the boot camp, being cancelled in order to reduce the risk of viral transmission between groups of students and clinicians. The boot camp could not be delivered in 2020 due to social distancing requirements. The cancellation of teaching activities had a significant impact on training, and in the face of adversity, many novel forms of education have come to the fore.^4^, ^5^, ^6^ One of the major changes to education has been the conversion of in‐person courses to virtual courses.

There are numerous positive aspects of virtual training that can be taken forward such as increasing technical and non‐technical skill acquisition through simulation.^7^, ^8^ It can also reduce the need for facilities and travel costs as well as improve the ease of attendance.^9^ Furthermore, virtual technology, such as the Proximie™ platform, can be used to enhance surgical education and can lead to a reduction in geographical barriers to medical education.^10^

The UK has come through the rapid wave of the Omicron variant of COVID‐19.^11^ Worldwide, millions of people remain in lockdown as outbreaks rise and fall across countries in a staggered fashion.^12^ In order to reduce the risk of transmission, social distancing, and therefore virtual courses, appear to be the safest strategies for the foreseeable future. In this prospective observational study, the methodology and experience of converting the urology boot camp for medical students into a virtual course is presented with key take‐home messages on how to convert an in‐person course to a virtual format. Results showing both quantitative and qualitative improvement in the knowledge and clinical acumen of course delegates are also presented.

## 2 METHODS

The course structure and core components, as used for the in‐person course, were maintained in the virtual course. The course was delivered annually in the autumn term, and this currently remains the case for the face‐to‐face course as well. The reason for this is that the timing is best suited for preparing students for medical school examinations in spring. All faculty had a background in medical education and had previously attended varying levels of formal teaching courses. The content of the teaching was based on the accompanying course textbook.^13^ Although no formal training on online teaching was given, it was on account of the ingenuity and adaptiveness of faculty that face‐to‐face teaching skills could be translated into online education.

Microsoft (MS) Teams™ was used as the virtual platform for the course. Baseline poll questions, using online polling software, were first asked of the delegates relating to how much of the urology curriculum had been covered by teaching sessions organized by their medical school thus far. Scores were acquired, using a 5‐point scale, for perceived knowledge of urology, confidence in the ability to perform urological clinical examinations and how well‐prepared delegates felt for medical school final examinations.

**FIGURE 2:**
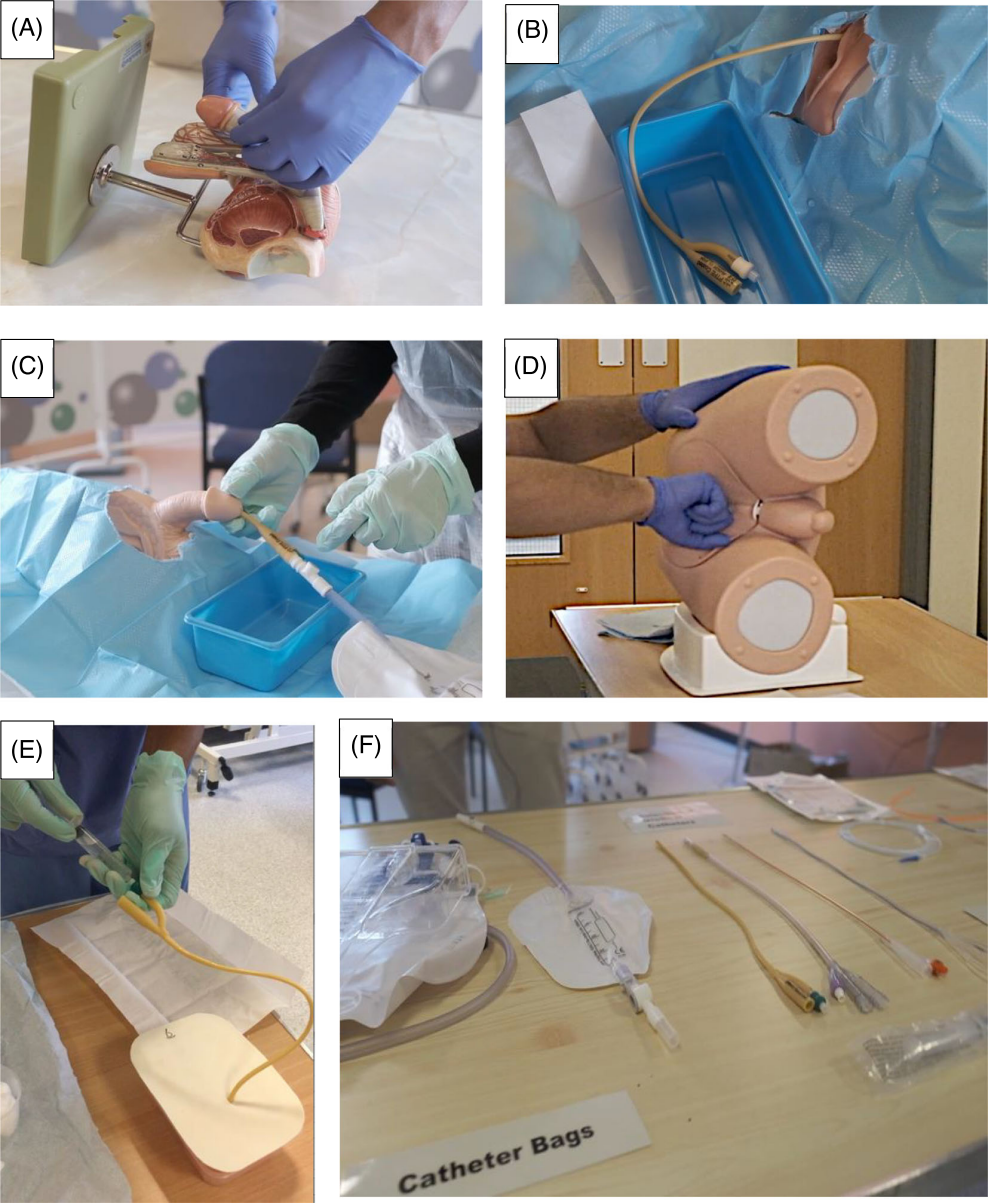
Screenshots taken from video‐recorded demonstrations using training models for; (A) external genitalia examination, (B) female catheterization, (C) male catheterization, (D) digital rectal examination, (E) routine exchange of a suprapubic catheter and (F) the use of common equipment in urology.

Videos were created by course faculty prior to the course to demonstrate the practical skills. These videos included male and female catheterization, digital rectal examination, external genitalia examination, routine change of a suprapubic catheter and the use of common equipment in urology (Figure 2). Video length was 5 min 18 s on average. This was in line with the principles outlined by Guo et al. in that shorter videos are more engaging.^14^ In their study of 6.9 million video‐watching sessions within four edX Massive Open Online Courses (MOOCs), it was found that longer videos should be segmented into parts that do not exceed a runtime of 6 min. In fact, for videos of 9‐ to 12‐min duration, engagement was shown to drop down to approximately 50%. The most popular YouTube medical education channel with over 2.5 million subscribers currently (29/11/22) is ‘Osmosis’, and the average video length on the channel is 6.9 min.^15^

The videos were played during the virtual course with a live running commentary and discussion between members of the course faculty. Delegates were encouraged to ask questions while the videos were played so that videos could be paused and played back to further explain pertinent points. Again, this was in line with the principles outlined by Guo et al.; providing a running commentary and allowing discussion during video sessions reduced cognitive load while maintaining learner engagement and promoting active learning. Although clinical skills such as catheterization were taught virtually, students were encouraged to visit local education departments and also to shadow ward doctors in order to gain ‘real‐world’ exposure to these practical tasks.

Didactic lectures were delivered live and these covered the theory‐based areas of the curriculum. The ‘chat’ function of the MS Teams™ platform was utilized throughout the course, with a member of the faculty designated to field through questions that delegates asked during the programme. This ensured that the speaker could focus on the lecture while questions could either be answered in the chat section for all to see, or vetted and put forward to the speaker to answer in real time. In addition, resources related to the presentation content were uploaded for delegates to refer to at a later date such as the British Association of Urological Surgeons (BAUS) consent forms as well as National Institute of Clinical Excellence (NICE) and European Association of Urology (EAU) guidelines. Slido™ was used for polling questions throughout the course. The duration of each lecture was segmented into 20‐min chunks to allow for frequent breaks and enable delegates to maintain concentration. Both in our experience and in the literature, this is the ideal lecturing time.^16^, ^17^

A Multiple‐Choice Questionnaire (MCQ), with a maximum score of 15, assessing the delegates' knowledge of urology according to the undergraduate curriculum, was designed. Face, construct and content validity were confirmed with assessment of the MCQs by urology clinicians and testing on a cohort of medical students. The MCQ required two iterations before the finalized version was confirmed, and Slido™ was used to implement the MCQ. The validated MCQ was used as a diagnostic assessment of prior knowledge before the course and as a formative assessment of learning after the course. Following the post‐course MCQ, faculty worked through the model answers with course delegates to maximize learning and shared understanding. A paired *t* test assuming equal variance was used to compare anonymized scores before and after the course to assess for statistically significant improvement as a measure of learning and course effectiveness. SPSS software version 26 (IBM SPSS Statistics for Mac, version 26, IBM Corp., Armonk, NY, USA) was used for data analysis.

On completion of the virtual course, delegates were asked how they rated the course on a 5‐point scale and whether they felt it improved their knowledge and confidence in urology. Delegates were also asked if they felt that the course prepared them for medical school final examinations and working as a foundation doctor. Open‐ended questions were asked of delegates relating to particular highlights of the course as well as how the course could be improved. An inductive thematic analysis was conducted in line with Braun and Clarkes' six‐phase framework.^18^ Microsoft Excel was used to tabularize data and colour‐code themes. These coded themes were then identified, reviewed and defined in order to apply to and optimize future iterations of the course.

At the end of the course, faculty debriefed to go through areas of the programme that went well and areas which could be improved. This was also a time to reflect on how each member of the faculty could develop their skills in training. In this way, faculty were able to continually improve their teaching skills with each implementation of the course.

## 3 RESULTS

**FIGURE 3:**
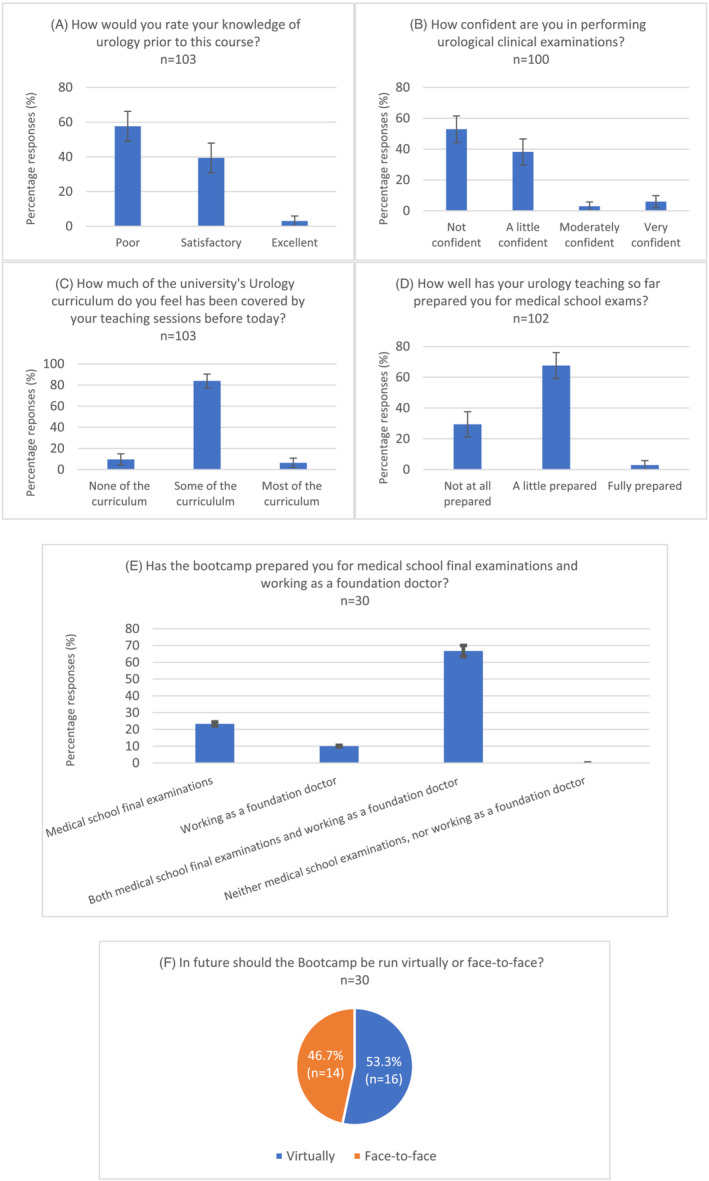
Delegates' responses to pre‐ and post‐course poll questions

As shown in Figure 3A,B, prior to undertaking the course, over half of delegates felt both that their knowledge of urology was ‘poor’ and that they were ‘not confident’ in performing urological clinical examinations. Less than 10% felt that most of the urology curriculum had been covered by their teaching sessions thus far (Figure 3C), and less than 5% felt that their urology teaching had fully prepared them for their medical school examinations (Figure 3D).

A total of 131 delegates took part in the virtual course in the year 2021, which constituted 40% (131/330) of the total final year (Year 5) group from the University of Nottingham Medical School. Of these, 105 delegates undertook the 15‐point pre‐ and post‐course MCQ. The mean score pre‐course was 7.12 (*SD* = 2.4) (47.5%), and at post‐course, this increased to 9.87 (*SD* = 2.45) (65.8%), showing a statistically significant 18.3% improvement following the course (*p* = <0.001).

For the most recent implementation of the virtual course, an overall course rating of ‘excellent’ was given by 63.3% (*n* = 19) of all delegates. All delegates (100%) felt that the boot camp improved their knowledge and confidence in urology. As shown in Figure 3E, all delegates felt that the course prepared them for medical school final examinations and working as a foundation doctor, with 66.7% (*n* = 21) reporting that it prepared them for both. Just over half of the delegates believed the course should be run virtually in the future instead of face‐to‐face (53.3% [*n* = 16]) (Figure 3F).

Thematic analysis of feedback received is shown in Table 1. Four themes for positive feedback were identified, which can be expanded upon and brought forward to optimize future iterations of the course. The Slido polling was received well with particular reference made to how it improved audience participation. The interactive nature of the course was highlighted as a uniquely positive aspect. Teaching quality was reported as being particularly good with appreciation of the different teaching styles used. Delegates also valued the fact that the depth of information was pitched at the right level. Positive comments were given pertaining to the content of the teaching delivered with importance placed on the relevance to the final year medical student curriculum and ultimately to working as foundation year one doctors. The course programme was reported as being ‘well‐organized’, and the various subsections of teaching were delivered in a clear and concise manner. Videos were also of particular value with delegates valuing the explanatory commentary to the filmed practical skills. All of these components will be maintained and further developed for future iterations of the course.

**TABLE 1:** Results of thematic analysis on post‐course delegate feedback with themes, sub themes and number of repetitions within the feedback

Positive feedback				
Theme	Sustained interactivity with attendees	High teaching quality	Appropriate depth of knowledge and skills covered	Well‐structured course
Subtheme	Use of Slido polling software	Use of instructional videos with a live running commentary	Course content tailored towards final year medical students	Concise and well‐organized
		Clarity of lectures		High yield
Number of repetitions	18	14	13	5

Ways to improve				
Theme	More case‐based discussions	Provision of supplementary learning material		
Number of repetitions	4	3		

Feedback on ways to improve the course included the provision of handouts. There were also requests for more case‐based discussions, and these seemed to be of particular interest to the students. In future, reference will be made before commencement of the course to the free textbook that accompanies the course and provides a framework especially for the lecture‐based teaching. Case‐based discussion will also be included as a formal part of the programme.

## 4 DISCUSSION

The urology boot camp for medical students was originally designed as a formative course, and this remains the case. The poll questions and MCQ assessment are diagnostic, allowing delegates to identify their weak areas and focus on areas for learning.

Delegates attend the course free of charge, and this is in alignment with the ethos of the course organizers that medical education should be freely available for those who wish to learn. The course is implemented with the support of medical education departments as well as faculty who voluntarily give their time to teach and develop their own training skills. Resources recommended on the course are freely available online. The accompanying course textbook, titled ‘Urology for medical students and junior doctors’, has an optional nominal fee, all profits of which go to funding medical education charities.^13^ This means that the boot camp runs with little to no extra financial costs for either setup or delivery. Utilizing resources in this way incurs no financial cost to delegates and allows attendance with no monetary cost.

The boot camp provides the opportunity for delegates to work in groups and engage in collective learning. Through this, they can build upon concepts learned previously and apply the theory‐based knowledge to clinical cases. The course employs Bruner's theory of spiral learning, allowing delegates to constructively learn, revisit topics and solidify key concepts.^19^ The formative polling questions throughout the course keep delegates engaged while providing a further medium for testing knowledge. The course also utilizes the ‘flipped classroom’ approach by encouraging learners to do their own research on topics and then apply it to problem solving, thereby being more applicable to clinical practice.^20^ At the end of the boot camp, reference is made to the course textbook.^13^ It is an invaluable resource that was written by the course organizers and can be used to consolidate learning in one's own time. In this way, the course provides a comprehensive educational experience providing assessment *for* learning and assessment *of* learning, using evidence‐based teaching methods.

The course is not just beneficial for delegates but also provides individualized continual feedback and training for faculty as medical educators. This has continued in the virtual format with designated time after the course for faculty to debrief together and discuss what worked well and areas for improvement. In this way, faculty continuously develop their training skills.

In this study, we demonstrate the successful conversion of the face‐to‐face urology boot camp for medical students to a virtual format. A number of measures were taken to maintain engagement during the virtual course and prevent screen fatigue. These included the use of online polling software, using chat functions to increase interactivity and also using video‐based learning. The added MCQ has now provided quantitative data on its effectiveness, showing a significant improvement in test scores after the boot camp. Delegate satisfaction results have remained overwhelmingly positive after the conversion to an online platform; Table 2 summarizes some pertinent quotations taken from delegate feedback.

**TABLE 2:** Quotations of positive delegate feedback

‘I thought it was an excellent overview of urology, in a short time frame! It was interactive and I liked the demonstration videos for clinical skills. I found it very helpful doing the quiz before & after. I thought it was a great revision day and feel more confident now with urology.’
‘Incredible teachers, who've created an incredibly well‐structured programme. Really broke down topics I often struggle to understand the theory behind. Lots of high yield knowledge for both finals and clinical practice. Also, love the videos! Overall, so so grateful you ran this session.’
‘Practical tips and clear and simple and concise explanation of why certain management and investigations are done. Very well organised! Giving information at the right level.’

A number of lessons have been learned from converting to an online platform. Top tips are summarized in Table 3 for any clinicians wishing to convert their own courses to an online format. The use of MS Teams™ enabled speakers to deliver live lectures to a large number of delegates with a high‐quality audio‐visual interface. However, other platforms such as Zoom™ have been described in the literature with excellent results.^21^ Giving a live running commentary to the videos maintained engagement and fostered a more interactive learning environment. The Slido™ software was chosen for polling and quiz questions on account of it being both user friendly and able to store data from questions for later analysis. Other polling platforms such as Kahoot!™ have also been described with positive outcomes.^22^

**TABLE 3:** Top tips for converting a face‐to‐face course to a virtual course

Engagement	Keep lecturing time to a minimum to reduce screen fatigue
	Have the audience keep their videos on to encourage active participation
	Include participant prizes at the end of the course to encourage engagement
	Ensure regular and more frequent breaks than an in‐person course
Delivery	Ensure participants keep microphones off to remove background noise
	Use a mix of media including audio, short pre‐recorded videos, live chat and video lecturing
	Select an online platform that is familiar to faculty, user‐friendly and cost‐effective
Interaction	Use interactive polls to increase interaction and enhance concentration
	Have one faculty member facilitating the ‘chat’ function and uploading resources
Faculty development	Assess the effectiveness of teaching by anonymous feedback and the assessment of learning
	Debrief after the course to discuss what worked well and areas for improvement

Boot camps are defined as compact, high‐intensity courses, which provide students with a foundation of curricular practical training for a new clinical role.^23^ With reducing opportunities for clinical students to gain practical experience and increasingly complex health needs, medical education boot camps are gaining popularity rapidly.^24^ The gap between final year of medical school and working as a doctor has always been a challenging transition. Virtual as well as face‐to‐face boot camps can be a solution to ease difficulties in this phase of training.^25^ The boot camp teaching style has been used in a number of different medical specialties including orthopaedic surgery, neurosurgery, emergency medicine and general internal medicine.^26^, ^27^, ^28^, ^29^, ^30^ These have all produced excellent results, not only in increasing the confidence of medical students to undertake their final examinations and future rotations in the specialty but also in inspiring them to join the various specialties. Boot camps offer the opportunity to network with clinicians, find mentors and learn more about a specialty career pathway.

In the UK, newly appointed urology registrars take part in the, now mandatory, registrar boot camp where delegates receive training in the core skills and knowledge required for starting work as a urology registrar.^31^ The strengths of the medical student boot camp are similar to the registrar boot camp such as its intensive, high‐yield teaching style and use of simulation.^32^ The registrar course has been well‐received by trainees with overwhelmingly positive feedback and a statistically demonstrable improvement in competence.^33^ It is likely that the medical student boot camp will continue to run in parallel with the registrar boot camp, following on from its successes in innovation and ingenuity.

Urology is at the forefront of integrating simulation with surgical training, with the first international randomized control trial assessing the transferability of urological simulation teaching currently underway.^34^ The use of virtual reality, fully immersive and cadaveric simulators as well as webinars and traditional dry lab models have all been reported in urological training with promising results.^35^, ^36^ As the urology boot camp for medical students continues to incorporate these new technologies, it is hoped that students will be further enabled to progress rapidly in their knowledge and skill acquisition.

UK undergraduate assessment and standards vary substantially between medical schools, and this is especially true for urological education.^1^, ^37^ Standardization brings equity, validity and fairness to medical education, which also reassures the public on the competency of graduates.^38^ The use of virtual training breaks down geographical barriers to education and enables access to high‐quality teaching. Incorporating virtual training into medical school curricula has the potential to bring standardization to undergraduate education.^39^ Just as the boot camp for urology registrars has enabled newly appointed trainees to be benchmarked against a national standard, the virtual and in‐person boot camps for medical students could potentially optimize undergraduate urological education across medical schools to meet the national standard.^40^

## 5 FUTURE DIRECTIONS

Following the success of the pilot virtual boot camp for medical students, the virtual format of the boot camp was successfully upscaled and expanded to reach more students. In October 2021, the course was delivered to an international audience of medical students and junior doctors. Delegates from more than 16 UK medical schools and 4 continents were able to attend the virtual boot camp without any financial cost. The course was rated as ‘excellent’ by 73% and a further 23% of delegates rated it as ‘very good’. Our plan is to continue to deliver this international course on an annual basis to improve access to teaching in urology and also reduce geographical and financial barriers to learning.

While the virtual course has transformed and advanced the delivery of the boot camp, the in‐person course will continue so as not to lose the vitally important hands‐on aspects of surgical training. Indeed, when students were asked whether they preferred in‐person or virtual courses, the split was roughly equal (Figure 3F), highlighting the need for both. The face‐to‐face course will run in parallel with the virtual course to ensure that the benefits of virtual learning are not lost while also maintaining practical skills training using simulation models. This will also enable Objective Structured Clinical Examinations (OSCEs) for delegates who attend face‐to‐face. Combined with the MCQs, this would provide more comprehensive preparation for final examinations as well as for working as competent foundation year one doctors.

Transitions through the levels of seniority in a medical career often come with new and unique challenges; the step from medical student to year one doctor is no exception.^41^ These transitions can be re‐framed as ‘critically intensive learning periods’ (CILPs).^42^ CILPs are times when trainees are particularly engaged and focused on obtaining the knowledge and skills to work in a particular environment. Providing structured, hands‐on, practical teaching during these times can overcome the challenges of working in new environments.^43^ In this way, boot camps can ease the challenges associated with working as a newly qualified doctor.

## 6 STRENGTHS AND LIMITATIONS

An inevitable limitation of the boot camp being delivered in a virtual format is that it is difficult to assess delegates competency in clinical skills and subjective measures of confidence do not always equate to clinical competence. Hands‐on, in‐person training is vital to surgical education, and this is why the face‐to‐face course will continue to run in parallel with the virtual course.

It will also be of value to study the long‐term learning assessment outcomes of the delegates by comparing cohorts who have not taken part in the boot camp to cohorts who have attended, with regard to how they perform in final medical school examinations and in clinical practice. Records have been kept of the students in each year group who did and who did not attend the boot camp, be it in its virtual or face‐to‐face format. It is planned to carry out a cohort comparative study to assess for difference in the level of urological skills and knowledge of ward doctors with those who attended the boot camp compared with those who did not. This will further elucidate the impact of the urology boot camp on clinical acumen.

One of the benefits of virtual platforms is that courses can be delivered nationally or internationally with relative ease. It may well be that boot camps of the future use a hybrid of both virtual and face‐to‐face interaction. This will likely develop as new technology in virtual reality and simulation are adopted. This could retain the elements of the course that are served best through physical meeting whilst maintaining the benefits of remote learning.

## 7 CONCLUSIONS

The urology boot camp for medical students has now received support from BAUS. As it continues to expand, it is hoped that it will not only bring standardization to undergraduate urology education but also inspire the next generation of doctors to embark on a career in urology. As virtual technology continues to be used to enhance medical education, the boot camp will remain at the forefront of these advancements.

## CONFLICT OF INTEREST

The authors declare no conflicts of interest.

## AUTHOR CONTRIBUTIONS

RE supervised course development and study design and also edited the manuscript. TF collected and analysed the data and drafted the manuscript. TF, MLH, CE and AG were all involved in course design, data collection and editing of the manuscript.
